# Routine healthcare disruptions: a longitudinal study on changes in self-management behavior during the COVID-19 pandemic

**DOI:** 10.1186/s12913-023-09119-x

**Published:** 2023-02-24

**Authors:** Juliane Menting, Femke van Schelven, Claire Aussems, Monique Heijmans, Hennie Boeije

**Affiliations:** Netherlands Institute for Health Services Research (Nivel), Utrecht, the Netherlands

**Keywords:** Chronic disease, Self-management, Adults, COVID-19, Access to health care, Mental health, Social support

## Abstract

**Background:**

The outbreak of COVID-19 had a significant impact on routines and continuity of professional care. As frequent users of this professional care, especially for people with chronic diseases this had consequences. Due to barriers in access to healthcare, an even greater appeal was made on the self-management behaviors of this group. In the present study, we aim to investigate the extent to which self-management changed during the recent pandemic, and which factors contributed to these changes.

**Methods:**

The Dutch ‘National Panel of people with Chronic Illness or Disability’ was used to collect self-reported data of people with at least one chronic disease. Self-management was assessed with the Partners in Health questionnaire at two time points: before the crisis in 2018 and during the second wave of crisis in Autumn 2020. Paired t-tests were used to analyze changes in self-management. Potential associating factors on three levels – patient, organization and environment – were assessed in 2020 and their impact on self-management changes was tested with multinomial logistic regression.

**Results:**

Data from 345 panel members was available at two time points. In the majority of people, self-management behaviors were stable (70.7%). About one in seven experienced improved self-management (15.1%), and a similar proportion experienced deteriorated self-management (14.2%). Sex, physical disability, mental health and daily stressors due to COVID-19 (patient level), changes in healthcare access (organization level), and social support (environment level) were significantly associated with experienced changes in self-management.

**Conclusions:**

People with chronic diseases experienced different trajectories of self-management changes during COVID-19. We need to be aware of people who seem to be more vulnerable to a healthcare crisis and report less stable self-management, such as those who experience mental health problems or daily stressors. Continuity of care and social support can buffer the impact of a healthcare crisis on self-management routines of people with chronic diseases.

## Background

The outbreak of COVID-19 had a significant impact on routine care within the Dutch healthcare system. Among others, this was caused by giving priority to acute care for patients with a COVID-19 infection and measures taken from the government to minimize the spread of the virus. Both factors caused delays in routine care. Especially people with one or more chronic diseases had to deal with healthcare related disruptions as they are frequent users of professional care. During the pandemic, they faced barriers in access to the healthcare system and healthcare utilization, such as cancelled or postponed medical appointments or deployment of telemedicine [1, 2]. In addition, measures of social distancing and quarantine resulted in disruptions in lifestyle, social life and possibilities for self-management and affected physical and psychosocial wellbeing [1].

Self-management is crucial in dealing with a chronic disease and achieving positive health outcomes. Self-management is the day-to-day management of a chronic disease by the individual over the course of an illness, and contains behaviors such as being physically active, keeping a healthy diet, being in contact with healthcare professionals and adhering to the diseases’ treatment plan and medication usage [3]. Especially patients with chronic diseases face these self-managements tasks and are required to take actions in managing their disease and treatment [4].

Disruptions in routine healthcare during a crisis such as the COVID-19 pandemic can have consequences for the balance between professional care and patients’ self-management. For some, the shift towards telemedicine contributed to improved self-management, as it increased flexibility in scheduling appointments with care providers [1]. Working from home and less obligations with family and friends may have led to more time and flexibility to plan self-management routines such as the regulation of medication, physical activity and the preparation of healthy meals. For others, the limited access to healthcare and support from healthcare professionals may be challenging and result in deteriorated self-management. Patients receive less support in their self-management and at the same time have to adapt to a changed daily life and new routines in managing their chronic disease [5, 6].

It is suggested that several factors affect changes in self-management behavior during the COVID-19 pandemic, both in a positive and negative way [2, 5, 7]. However, this has been hardly studied so far. Literature published before the pandemic has extensively described factors on different levels influencing self-management, such as patients’ physical and mental health [8, 9], accessibility to healthcare and information, shared decision making between patient and professionals [9–11], and family and peer support [3, 8, 12]. It is likely that these factors, related to the patient, healthcare organization and environment of the patient, also affect changes in self-management during a healthcare crisis in which routine healthcare is disrupted, such as the COVID-19 pandemic. For example, research has shown that poor mental health is associated with avoidance of routine medical visits and difficulty in managing health and medications during the pandemic [13, 14]. Low literacy may also be related to negative changes in self-management behavior during the pandemic, due to difficulties in adapting to telemedicine [1, 15]. Other factors that may contribute to changes in self-management during the pandemic are patients’ sex [2, 13], age [15], education [2] social support [16] and a lack of professional support in self-management [6].

So, although the literature provides some clues that changes in self-management during a pandemic like COVID-19 are influenced by different factors on the patient, healthcare organization and environmental level, little research has actually focused on this question. The present study aims to investigate the extent to which self-management changes during a crisis in which routine healthcare is disrupted, and which factors contribute to these changes, by answering the following research questions:

1. Do changes in self-management behaviors between the pre-COVID-19 period and during the COVID-19 pandemic take place in people with a chronic disease, and, if so, to what extent?
2. Which factors on the patient, organizational and environmental level influence a person’s change in self-management behavior?

## Methods

### Procedure and participants

The research was part of the Dutch nationwide panel ‘National Panel of people with Chronic Illness or Disability’ (NPCD). Since 1998, the NPCD reports about the experiences with and consequences of living with a chronic condition or long-term physical disability from the patients’ perspective. The panel consists of approximately 3500 people aged ≥ 15 years who are recruited from a random sample of general practices in the Netherlands. Within the selected practices, general practitioners (GPs) are invited to participate in the selection of patients with a chronic disease. With help from trained research staff, participating GPs draw a random sample constituting one third of their patients. All patients who are diagnosed with one or more somatic chronic diseases according to the International Classification of Primary Care (ICPC) are eligible for inclusion [17]. Exclusion criteria are: < 15 years old, being institutionalized, unaware of diagnosis, being terminally ill (a life expectancy of < 6 months) and insufficient mastery of the Dutch language. Included patients receive a letter from their GP with information about the panel, an invitation to participate and an informed consent form. After subscribing for the NPCD, patients receive a short questionnaire to provide some background information, such as gender, age and education. After returning this questionnaire, a patient becomes member of the NPCD. New panel members are selected annually to replace participants who withdrew or had participated for the maximum term of four years.

Twice a year, i.e., in April and October, panel members receive a postal or online questionnaire on various topics including health-related outcomes, use of and experiences with healthcare and experiences with participation in society. For the purpose of the present study, we used data collected in October, 2018 and 2020. We selected only panel members with one or more chronic somatic conditions. The NPCD is representative of most common chronic diseases such as cardiovascular diseases, diabetes, cancer, musculoskeletal diseases or chronic obstructive pulmonary diseases. Between October and November, 2018, 2484 panel members with one or more chronic diseases were invited to answer questions on self-management. A total of 1857 panel members filled in the questionnaire (response rate = 75%). In the period between October and November, 2020, 1713 out of 2179 panel members filled out questions about self-management again, together with questions on the potential associating factors (response rate = 79%).

At the time of the second measurement point in autumn 2020, there was a second wave of COVID-19 infections in the Netherlands and containment measures were implemented. First, a partial lockdown was ordered with measures consisting of social distancing, restaurants closing early and closing of museums and swimming pools. Later on in that period, the lockdown was expanded with extensive measures such as closing schools, sport facilities and non-essential stores, and working from home [18].

### Measures

#### Self-management

Perceived self-management was assessed with the Partners in Health (PiH) questionnaire [19]. The PiH consists of 12 items and measures patients’ chronic condition self-management knowledge and behaviors. It contains questions about, for example, the knowledge of the disease and its treatment, the ability of a person to monitor and manage disease symptoms and the extent to which a person plays an active role in consults with care providers. Each item is scored from zero to eight with higher scores indicating better self-management. The internal consistency of the PiH in this study was good with Cronbach’s alpha of 0.84, both in 2018 and 2020.

#### Potential associating factors of self-management

All factors potentially related to perceived self-management behavior are chosen in consistence of earlier research and literature mentioned above.

#### Factors on the patient level

##### Personal characteristics and health status

Data on socio-demographic variables and disease-status were collected when respondents entered the panel. We looked at sex (male, female), age (continuous variable) and educational level (low, middle and high) for socio-demographic variables. Health status was indicated with the self-report measurement for severity of physical disability due to the chronic disease (no disability, mild disability, moderate disability, severe disability) [17].

*Mental health* was assessed with the dimension anxiety/depression of the EQ-5D questionnaire [20]. The EQ-5D is a commonly used and validated instrument for health measurement. In addition to its measurement of the patients’ general health status, it can evaluate patients’ health and functioning on five dimensions: mobility, self-care, usual activities, pain/discomfort, and anxiety/depression. The anxiety/depression dimension is scored on a five-point Likert Scale from 1 ‘I am not anxious or depressed’ to 5 ‘I am extremely anxious or depressed’. For the present research, we used a dichotomous variable for anxiety/depression (‘none or very limited problem’ vs. ‘moderate to severe problem’).

*Daily stressors due to COVID-19* were determined by asking respondents whether they experienced one or more consequences in their daily lives due to the COVID-19 pandemic: (1) having to stay at home or work from home as much as possible, (2) having health concerns about themselves or significant others, (3) having less social contacts, (4) being less able to do leisure activities, and (5) being afraid or experiencing tension that others do not keep enough distance. The checked response options were computed by counting the number of daily stressors (0–5).

#### Factors related to the organization of care including the patient-provider interaction

*Changes in access to care due to COVID-19* were assessed by asking respondents whether the COVID-19 pandemic affected the professional healthcare or support they received for their chronic disease. Response options were ‘no’ and ‘yes’.

*Shared decision making* and *information provision* were measured with the Patient Reported Experience Measure (PREM) Chronic Care [21]. The PREM Chronic Care is a validated Dutch questionnaire that measures patients’ experiences with different aspects of chronic healthcare on 14 items. Each item is scored on a five-point Likert scale from 1 ‘not at all’ to 5 ‘very much’. For the present study, items on shared decision making (‘together with the healthcare professional, I can discuss how I want to work on my health’) and information provision in the course of the disease treatment (‘the healthcare professional gives me information on what I can do if my symptoms change’) were used.

#### Factors on the environmental context

*Social support* was indicated with the subscale social loneliness of the De Jong Gierveld loneliness questionnaire [22]. This questionnaire is an often-used and validated measure. The subscale social loneliness consists of five items (e.g., ‘There is always someone I can talk to about my day-to-day problems’) with each item scoring on ‘no’, ‘yes’, or ‘more or less’. All items were summed and categorized (‘no social support’ vs. ‘social support’).

The socio-demographic variable *living situation* (living alone or with others) was assessed when patients entered the panel.

### Statistical analysis

Statistical analyses were performed using Stata 15.0 [23]. We hypothesized that perceived self-management between 2018 and 2020 has changed differently between people, and divided respondents into three groups based on the mean and standard deviation of the PiH change score (range of PiH change score = -2.92 – 2.83): 1) improved self-management (0.83 – 2.83), 2) unchanged self-management (-0.85 – 0.83), and 3) deteriorated self-management (-2.9 – -0.85). In this process, one extreme outlier (with a mean PiH change score of -8) was removed from the data. For all three groups, differences in self-management scores between 2018 and 2020 were tested with paired t-tests.

Multinomial logistic regression was performed to test the relationship between factors on personal, organizational and environmental level that are potentially related to a person’s change in self-management. The three self-management groups were set as outcome variable whereas stable self-management was used as reference group (improved vs. stable management, deteriorated vs. stable self-management). The factors were added stepwise to the model, based on literature findings and models related to self-management. Model 1 (personal characteristics and health status) contained the factors sex, age, educational level, severity of disability, mental health, and daily stressors due to COVID-19. In model 2 (healthcare system), changes in access to care due to COVID-19, and experiences with patient-provider interactions such as shared decision making and information provision were added. In model 3 (environmental context), living situation and social support were added. *P*-values < 0.05 were regarded as significant.

## Results

From a total of 345 panel members, data on both measurement points (2018 and 2020) were available. About half of the respondents was female (*n* = 179, 51.9%) and the mean age in 2020 was 67.1 years (*n* = 345, SD = 12.0). Most common chronic diseases were asthma and chronic obstructive pulmonary diseases (*n* = 59, 17.1%), cardiovascular diseases (*n* = 59, 17.1%), diabetes mellitus (*n* = 53, 15.4%), and musculoskeletal diseases (*n* = 52, 15.1%). The majority of respondents were living with a spouse or partner (*n* = 240, 70.4%), and had no or mild physical disability due to their chronic disease(s) (*n* = 220, 66.9%). Low, middle and high educational level was 19.9% (*n* = 67), 47.3% (*n* = 159) and 32.7% (*n* = 110), respectively. Table 1 provides an overview of the percentages and means on the potential associating factors with self-management that were studied.

**Table 1:** Percentages and means on potential associating factors of self-management, as reported by participants in 2020

	n	%
Sex		
Female	179	51.9
Male	166	48.1
Age (mean(SD))	345	67.1
Educational level		
Low	67	19.9
Middle	159	47.3
High	110	32.7
Severity of disability		
No	131	39.8
Mild	89	27.1
Moderate	85	25.8
High	24	7.3
Mental health problems		
None to very limited	310	90.1
Mild to severe	34	9.9
Daily stressors due to COVID-19 (mean)	341	2.4
Changes in access to care due to COVID-19		
Yes	116	33.9
No	226	66.1
Shared decision making (mean)	264	4.2
Information provision (mean)	264	3.9
Living situation		
With a spouse or partner	240	70.4
Without a spouse or partner	101	29.6
Social support		
Yes	212	62.9
No	125	37.1

### Changes in self-management

The majority of respondents reported unchanged self-management from 2018 to 2020 (*n* = 244, 70.7%). About one in seven experienced improved self-management (*n* = 52, 15.1%), and a similar proportion experienced deteriorated self-management (*n* = 49, 14.2%). For the improved self-management group, average PiH-scores increased from 2018 to 2020 with 1.27 points (*p* < 0.001). Mean scores of those who reported unchanged self-management were persistently high, with an approximate value of seven in both years (M_dif f_ = 0.01, *p* = 0.695). In the deteriorated self-management group, average PiH-scores decreased from 2018 to 2020 with 1.45 points (*p* < 0.001). The corresponding mean PiH-scores are presented in Table 2.

**Table 2:** Changes in self-management, PiH-scores of the three groups, 2018 and 2020

	PiH-score 2018		PiH-score 2020		PiH change score		
	M	SD	M	SD	M_diff_	SD_diff_	p-value
Improved self-management	5.81	0.79	7.08	0.71	1.27	0.41	0.000
Unchanged self-management	6.97	0.70	6.98	0.75	0.01	0.39	0.695
Deteriorated self-management	7.29	0.57	5.85	0.78	-1.45	0.47	0.000

*Notes.* *PiH*  Partners in Health questionnaire, range 0–8

M_diff_ = Mean of PiH change score, SD_diff_ = Standard deviation of change score

### Associations of improved and deteriorated self-management

The fit of model 1 was good with X2 (18, *N* = 317) = 30.11, *p* < 0.05. The overall fit of the other models was not significant with X2 (24, *N* = 242) = 35.99, *p* = 0.055 (model 2) and X2 (28, *N* = 238) = 41.04, *p* = 0.05 (model 3). Nevertheless, it was chosen to continue with these models as the literature suggests the value of the added variables in the models and the p-values were close to 0.05. The modeled variance increased slightly from model 1 to model 3 (R^2^ = 0.06, 0.09, and 0.11, respectively).

Table 3 presents the results of the regression models for the improved self-management group. From the factors on the patient level (model 1), severity of disability, mental health, and daily stressors due to COVID-19 were significantly associated with improved self-management. In comparison with the stable self-management group, respondents who experienced improved self-management had moderate severity of disability (β = 0.98, *p* = 0.016), had more mental health problems (β = 1.10, *p* = 0.045), and experienced less daily stressors due to the COVID-19 pandemic (β = -0.31, *p* = 0.036). From the factors related to the organization of healthcare (model 2), changes in access due to the COVID-19 pandemic were significantly related to improved self-management. In comparison to respondents from the stable self-management group, those in the improved self-management group experienced less often changes in their care due to the COVID-19 pandemic (β = -1.08, *p* = 0.026). In addition, educational level became a significant factor with improved self-management associated with lower educational level (β = 1.18, *p* = 0.024). From the environmental context factors (model 3), social support was associated with improved self-management with higher levels of social support seen in those with improved self-management compared to those with stable self-management (β = 1.18, *p* = 0.027). In addition, sex became a significant factor with improved self-management associated with males (β = -0.98, *p* = 0.034). Educational level and daily stressors due to COVID-19 were no longer significant in model 3.

**Table 3:** Multinomial logistic regression with changes in self-management (improved self-management) as dependent variable

	Model1 (*n* = 317) *Personal characteristics and health status*	Model2 (*n* = 242) *Healthcare system*	Model3 (*n* = 238) *Environmental context and support*
Intercept	-1.00 (1.04)	-1.50 (1.73)	-1.04 (1.82)
Sex (female)	-0.37 (0.34)	-0.55 (0.42)	-0.98 (0.46)*
Age (higher)	-0.01 (0.01)	-0.03 (0.02)	-0.03 (0.02)
Educational level^a^			
Low	0.81 (0.41)	1.18 (0.52)*	1.05 (0.55)
High	0.19 (0.41)	0.11 (0.51)	0.08 (0.52)
Severity of disability^b^			
Mild	0.10 (0.45)	0.77 (0.55)	0.75 (0.58)
Moderate	0.98 (0.41)*	1.27 (0.54)*	1.59 (0.58)**
High	-0.40 (0.83)	0.59 (0.95)	0.46 (0.98)
Mental health problems (mild to severe)	1.10 (0.55)*	1.38 (0.66)*	1.81 (0.71)*
Daily stressors due to COVID-19 (high)	-0.31 (0.15)*	-0.21 (0.17)	-0.18 (0.18)
Changes in access to care due to COVID-19 (yes)		-1.08 (0.42)*	-1.18 (0.51)*
Shared decision making (more)		0.49 (0.40)	0.42 (0.42)
Information provision (more)		-0.11 (0.32)	-0.19 (0.33)
Living situation (with others)			-0.59 (0.46)
Social support (yes)			1.18 (0.53)*
R^2^	0.06	0.09	0.11

*Notes.* Entries are coefficients with standard errors in parentheses. The reference category is unchanged self-management

^*^ *p* < .05, ** *p* < .01, *** *p* < .001

^a^ Middle education was used as a reference group

^b^ No disability was used as reference group

Table 4 shows the results of the regression models for the deteriorated self-management group. In the first model, sex and mental health were associated with deteriorated self-management. In comparison with the stable self-management group, respondents in the deteriorated self-management group were more often males (β = -0.73, *p* = 0.036) and those with mild to severe mental health problems (β = 1.45, *p* = 0.003). In the second and third model, no additional factors were found to be significant. Sex and mental health problems remained significantly associated to deteriorated self-management.

**Table 4:** Multinomial logistic regression with changes in self-management (deteriorated self-management) as dependent variable

	Model1 (*n* = 317) *Personal characteristics and health status*	Model2 (*n* = 242) *Healthcare system*	Model3 (*n* = 238) *Environmental context and support*
Intercept	-1.89 (1.01)	0.09 (1.58)	0.29 (1.63)
Sex (female)	-0.73 (0.35)*	-0.93 (0.40)*	-1.03 (0.42)*
Age (higher)	0.01 (0.01)	0.01 (0.02)	0.01 (0.02)
Educational level^a^			
Low	-0.05 (0.49)	0.03 (0.55)	-0.03 (0.55)
High	0.41 (0.37)	0.19 (0.42)	0.18 (0.42)
Severity of disability^b^			
Mild	0.20 (0.41)	0.17 (0.49)	0.20 (0.49)
Moderate	0.07 (0.45)	0.21 (0.52)	0.28 (0.53)
High	0.10 (0.65)	0.11 (0.78)	0.08 (0.78)
Mental health problems (mild to severe)	1.45 (0.48)**	1.30 (0.57)*	1.34 (0.60)*
Daily stressors due to COVID-19 (high)	-0.11 (0.14)	-0.07 (0.16)	-0.07 (0.16)
Changes in access to care due to COVID-19 (yes)		0.06 (0.41)	0.06 (0.41)
Shared decision making (more)		-0.26 (0.31)	-0.30 (0.32)
Information provision (more)		-0.31 (0.28)	-0.29 (0.29)
Living situation (with others)			-0.32 (0.45)
Social support (yes)			0.28 (0.42)
R^2^	0.06	0.09	0.11

*Notes.* Entries are coefficients with standard errors in parentheses. The reference category is unchanged self-management

^*^ *p* < .05, ** *p* < .01, *** *p* < .001

^a^ Middle education was used as a reference group

^b^ No disability was used as reference group

## Discussion

This research showed that perceived self-management behavior was stable among the majority of people with somatic chronic diseases between 2018 and the second wave of the COVID-19 pandemic. During this period, a (partial) lockdown was ordered in the Netherlands with measures consisting of social distancing, working from home, closing of museums and swimming pools and – later – closing of schools, sport facilities and non-essential stores [18]. Although the majority experienced no changes, perceived self-management behavior changed in three out of ten people with chronic diseases, displaying different change trajectories. About 15 percent experienced improved self-management behavior, 14 percent reported deteriorated self-management behavior.

The findings of the present study corroborate previous research on self-management during the COVID-19 pandemic. Based on our design we cannot pinpoint the exact cause for the perceived changes in self-management behavior, but it is likely that the pandemic played a role. Previous literature reviews also suggest that the pandemic influenced self-management behaviors among people with chronic diseases [1, 24, 25]. In qualitative and mixed-method studies, people with chronic diseases indicated themselves that they adapted their self-management routines during the pandemic, both in positive and negative ways [5, 26]. Similar findings were reported in quantitative studies that specifically ask participants about changes in self-management due to the pandemic [2, 15, 27]. This supports the assumption that the changes we demonstrated in the present study are at least to some extent the result of the COVID-19 pandemic.

To shed light on the different patterns of changes in perceived self-management behavior during the COVID-19 pandemic, we determined potential contributing factors. Importantly, we found that people who experienced no healthcare disruptions such as limited access to professional care reported increased self-management. The extent of shared decision making and information provision by care providers were not significantly correlated with changes in perceived self-management. Continuity of care appeared to be most important in the contact between people with chronic diseases and care providers during the COVID-19 pandemic. This corresponds with literature focusing on generic factors affecting self-management [9] and COVID-19-specific factors affecting self-management [14]. Continuity in appointments with their healthcare providers can support people with chronic diseases in adapting self-management behaviors during a time when existing selfcare routines are under pressure due to social distancing, quarantine and hospital restriction measures [1, 6]. However, it is precisely during a healthcare crisis such as the pandemic that continuity of care is under pressure. This highlights the importance of finding good alternatives for monitoring and supporting self-management, such as effective access to telemedicine for different kinds of patient groups [1] and interventions focusing on improving self-management skills [26].

Our results indicated that no or mild mental health problems is an important prerequisite for stable perceived self-management during the COVID-19 pandemic. Experiencing specific COVID-19 related stressors, such as being less able to do leisure activities, was found to have a negative impact on perceived self-management behavior, although this relation was no longer significant after adding the variables on the healthcare system and environmental context level. These findings are in line with a study of Lovett and colleagues [14], who found that deteriorated mental health and increased levels of stress during the pandemic are associated with greater self-reported difficulty in managing health and medications, more barriers to medication adherence and greater avoidance of healthcare visits. It is important to provide mental health support to people with chronic diseases, especially since they experience more anxiety and depression symptoms and levels of stress during the COVID-19 pandemic than those without chronic diseases [28, 29].

Previous studies have shown that a lack of social support during the COVID-19 pandemic is likely to exacerbate mental health problems [30], and thus patients’ ability to self-manage. Our study showed that social support cannot solely prevent deteriorated self-management during a crisis, but can also actually improve self-management. A possible explanation for this finding is that social support compensates for the limited access to and support from healthcare professionals during the pandemic. Family and friends can help people with chronic diseases to adopt new routines in managing their disease. This emphasizes the need for good alternatives for social contact in times of social distancing and quarantine.

Finally, we found that men experienced more changes in self-management than women. A possible explanation is the attitude in coping with illness. Whereas men tend to focus on practical aspects of self-management, it has been shown that women focus on affective components [31]. Possibly, men’s self-management behavior was affected by the influence the COVID-19 pandemic had on various practical aspects of self-management such as more flexibility in work or daily activities. Following this line of reasoning, it would have been likely to find changes in the self-management of women as well, due to measures such as social distancing and quarantine. However, we did not find this in our data. A possible explanation is that women used digital communication methods to replace social contacts. This assumption is supported by other studies showing that women and girls are more likely than men and boys to increase their communication with family and friends outside the household using digital communication methods [32, 33].

## Conclusions

The present study showed that perceived self-management behavior is variable in one out of three patients with a chronic disease during a healthcare crisis such as the COVID-19 pandemic. Especially those who experience mental health problems or stressors due to the crisis report to be less stable in the management of their chronic illness. Continuity of care and social support can help people with chronic conditions to adapt to new routines in self-management. It is vital that healthcare providers are aware of the factors affecting self-management during a healthcare crisis such as the pandemic and provide the right support, as this is crucial in regulating disease activities and achieving positive health outcomes**.**

### Strengths and limitations

The present study investigated perceived changes in self-management among a large group people with chronic diseases by comparing data before and during the COVID-19 pandemic. Data from the NPCD were used and the recruitment strategy within this panel is considered an important strength; they were selected from a nationwide random sample and their chronic disease is based on a GP’s diagnosis rather than self-report. There were however also a few limitations in our study design. First, the factors associated with changes in self-management behavior were measured at one timepoint (in 2020) and not available for the datapoint in 2018. Consequently, it was not possible to correct for potential changes in the independent variables. Second, the interval between the two measurement points was relatively long. It could be that self-management levels are more variable and change frequently over time. The results of the present study must be observed in the context of the COVID-19 infection rates and measures at the time of the second measurement in autumn 2020. At that timepoint, the infection rates were relatively high and containment measures taken were quite strict. It would be interesting to monitor self-management behavior after 2020 and in the future, to investigate how a long lasting crisis in healthcare, such as the COVID-19 pandemic, affects patients’ self-management behavior during different time points of a crisis. Finally, we used available data to study self-management changes and potentially contributing factors. It is, however, likely that even more factors are related to self-management changes, such as patients’ knowledge, motivation or beliefs about health and illness [9, 12]. It would be interesting for future research to study the potential impact of these factors on changes in self-management.

## Data Availability

The datasets used and/or analysed during the current study are available from the corresponding author on reasonable request.
